# Central centrifugal cicatricial alopecia: retrospective case-control study of 54 patients from a tertiary care center

**DOI:** 10.1097/JW9.0000000000000206

**Published:** 2025-05-01

**Authors:** Katherine L. Wang, Thais P. Pincelli, Ashley B. Wentworth, Launia J. White, Zhuo Li, Olayemi Sokumbi, Alison J. Bruce

**Affiliations:** a Mayo Clinic Alix School of Medicine, Mayo Clinic, Jacksonville, Florida; b Department of Dermatology, Mayo Clinic, Jacksonville, Florida; c Division of Clinical Trials and Biostatistics, Mayo Clinic, Jacksonville, Florida; d Department of Laboratory Medicine and Pathology, Mayo Clinic, Jacksonville, Florida

**Keywords:** alopecia, central centrifugal cicatricial alopecia, cicatrization, hair follicle, hair preparations, scarring alopecia

**What is known about this subject in regard to women and their families?**

- Central centrifugal cicatricial alopecia (CCCA) is a common scarring alopecia mainly affecting Black women.
- Despite its high prevalence, CCCA remains understudied.

**What is new from this article as messages for women and their families?**

- Patients with CCCA were significantly less likely to report improvement following treatment compared to controls with non-CCCA scarring alopecia.
- CCCA patients were significantly more likely to be treated with oral antibiotics and significantly less likely to be treated with oral 5α-reductase inhibitors and hydroxychloroquine.

Central centrifugal cicatricial alopecia (CCCA) is a scarring alopecia that predominantly affects women of African descent. With a prevalence of 2.7 to 5.6% in Black women, it is among the most common types of scarring alopecia.^1,2^ Numerous associations with CCCA have been described. A genetic basis for the development of CCCA has been postulated, with some cases noted to run in families and to display autosomal dominant inheritance.^3^ In a cohort of CCCA patients, Shah and Alexis^4^ found that all patients had a history of traumatic styling practices, including high-tension styles, chemical relaxers, and the use of heated styling tools. The aim of this study is to characterize the clinical features of CCCA patients at our institution and compare them to a control group of patients with non-CCCA scarring alopecia.

With institutional review board approval, we reviewed patients with CCCA and non-CCCA scarring alopecia from 2000 to 2022. Non-CCCA scarring alopecia included lichen planopilaris, frontal fibrosing alopecia, discoid lupus, acne keloidalis nuchae, folliculitis decalvans, dissecting cellulitis, and other unspecified cicatricial alopecia. All patients had biopsy-confirmed disease. For this study, “high tension hairstyles” included braids, cornrows, extensions, and tight ponytails; “heating tools” included curling irons, flat irons, and hot combs.

We identified 54 CCCA and 270 control patients (Table 1). Subjects with CCCA were significantly less likely to improve following treatment compared with controls (31.5% vs 59.6%; *P* = .0004). CCCA patients were more frequently treated with oral antibiotics (55.6% vs 33.0%; *P* = .0030) than with oral 5α-reductase inhibitors (1.9% vs 16.3%; *P* = .0023) or hydroxychloroquine (13.0% vs 37.8%; *P* = .0003). A positive family history of unspecified alopecia was more commonly reported in CCCA patients than controls (38.9% vs 14.8%; *P* = .0002). Use of high-tension hairstyles (46.3% vs 2.2%; *P* < .0001), heating tools (14.8% vs 3.0%; *P* = .0002), or chemical relaxants/perms/hair dyes (44.4% vs 6.7%; *P* < .0001) was more frequently indicated by CCCA patients versus controls (Table 2).

**Table 1:** Comparison of demographic and treatment data between CCCA and control patients

	Total (*N* = 324)	Central centrifugal cicatricial alopecia (*N* = 54)	Other form of scarring alopecia (*N* = 270)	*P* value
American Indian or Alaska Native, *n* (%)				1.0000^a^
	1 (0.3%)	0 (0.0%)	1 (0.4%)	
Asian, *n* (%)				0.2288^a^
	12 (3.7%)	0 (0.0%)	12 (4.4%)	
Black or African American, *n* (%)				<.0001^a^
	84 (25.9%)	49 (90.7%)	35 (13.0%)	
Native Hawaiian or other Pacific Islander, *n* (%)				1.0000^a^
	1 (0.3%)	0 (0.0%)	1 (0.4%)	
Other, *n* (%)				1.0000^a^
	4 (1.2%)	0 (0.0%)	4 (1.5%)	
Unknown, *n* (%)				1.0000^a^
	8 (2.5%)	1 (1.9%)	7 (2.6%)	
White or Caucasian, *n* (%)				<.0001^a^
	214 (66.0%)	4 (7.4%)	210 (77.8%)	
Family history of alopecia, *n* (%)				0.0002^a^
Yes	61 (18.8%)	21 (38.9%)	40 (14.8%)	
No	55 (17.0%)	10 (18.5%)	45 (16.7%)	
Unknown	208 (64.2%)	23 (42.6%)	185 (68.5%)	
Preceding general anesthesia, *n* (%)				1.0000^a^
Yes	4 (1.2%)	0 (0.0%)	4 (1.5%)	
No	320 (98.8%)	54 (100.0%)	266 (98.5%)	
Improvement following treatment, *n* (%)				0.0004^a^
Yes	178 (54.9%)	17 (31.5%)	161 (59.6%)	
No	43 (13.3%)	9 (16.7%)	34 (12.6%)	
Unknown	103 (31.8%)	28 (51.9%)	75 (27.8%)	
Topical steroids (class I), *n* (%)				1.0000^a^
	197 (60.8%)	33 (61.1%)	164 (60.7%)	
Intralesional steroids, *n* (%)				0.8708^a^
	97 (29.9%)	17 (31.5%)	80 (29.6%)	
Systemic steroids, *n* (%)				1.0000^a^
	5 (1.5%)	1 (1.9%)	4 (1.5%)	
Topical minoxidil, *n* (%)				0.0941^a^
	123 (38.0%)	26 (48.1%)	97 (35.9%)	
Oral minoxidil, *n* (%)				0.4794^a^
	14 (4.3%)	1 (1.9%)	13 (4.8%)	
Topical finasteride, *n* (%)				0.1952^a^
	5 (1.5%)	2 (3.7%)	3 (1.1%)	
Oral finasteride, *n* (%)				0.0023^a^
	45 (13.9%)	1 (1.9%)	44 (16.3%)	
Oral spironolactone, *n* (%)				0.0044^a^
	3 (0.9%)	3 (5.6%)	0 (0.0%)	
Topical JAK inhibitor, *n* (%)				0.6056^a^
	7 (2.2%)	0 (0.0%)	7 (2.6%)	
Oral JAK inhibitor, *n* (%)				1.0000^a^
	1 (0.3%)	0 (0.0%)	1 (0.4%)	
Oral biotin, *n* (%)				0.1324^a^
	8 (2.5%)	3 (5.6%)	5 (1.9%)	
Oral antibiotics, *n* (%)				0.0030^a^
	119 (36.7%)	30 (55.6%)	89 (33.0%)	
Oral hydroxychloroquine, *n* (%)				0.0003^a^
	109 (33.6%)	7 (13.0%)	102 (37.8%)	
Topical antibiotics, *n* (%)				0.1921^a^
	29 (9.0%)	2 (3.7%)	27 (10.0%)	
Other oral vitamin supplement, *n* (%)				1.0000^a^
	13 (4.0%)	2 (3.7%)	11 (4.1%)	
Other treatments, *n* (%)				0.0713^a^
	94 (29.0%)	10 (18.5%)	84 (31.1%)	
No treatment recommended, *n* (%)				0.0243^a^
	15 (4.6%)	6 (11.1%)	9 (3.3%)	

JAK, Janus kinase.

a Fisher’s exact *P*-value.

**Table 2:** Comparison of styling practices between CCCA and control patients

	Total (*N* = 324)	Central centrifugal cicatricial alopecia (*N* = 54)	Other form of scarring alopecia (*N* = 270)	*P* value
Preceding use of high-tension hair styles, *n* (%)				<.0001^a^
No	293 (90.4%)	29 (53.7%)	264 (97.8%)	
Yes	31 (9.6%)	25 (46.3%)	6 (2.2%)	
Preceding use of heating tools, *n* (%)				0.0002^a^
No	308 (95.1%)	46 (85.2%)	262 (97.0%)	
Yes	16 (4.9%)	8 (14.8%)	8 (3.0%)	
Preceding use of chemical relaxants, perms, or hair dyes, *n* (%)				<.0001^a^
No	282 (87.0%)	30 (55.6%)	252 (93.3%)	
Yes	42 (13.0%)	24 (44.4%)	18 (6.7%)	

a Chi-square *P* value.

In this study, clinical features of CCCA were characterized and compared to controls with non-CCCA scarring alopecia. One potential reason for the reduced likelihood of improvement in CCCA patients is the variation in treatment choices. Another possibility could be the genetic component of CCCA, which may alter the response to treatment. The genetic predisposition in CCCA can influence disease progression and severity, portending less responsiveness to conventional therapies. Such genetic factors may affect the follicular capability for regeneration and response to treatment, leading to less favorable outcomes compared with other types of scarring alopecia. The greater prevalence of a positive family history in CCCA patients compared with controls emphasizes the significance of genetic predisposition in the development of this condition. Although our study found higher rates of high-tension hairstyles, heating tools, and relaxants/perms/hair dyes among CCCA patients, this may be attributed to the fact that CCCA is more prevalent in Black patients, who are more likely to use these hairstyling techniques.^5^ Limitations of the present study include retrospective analysis, data from a tertiary referral center, and limited long-term follow-up. Future studies may further characterize treatments and outcomes in CCCA versus non-CCCA scarring alopecia patients.

## Conflicts of interest

None.

## Funding

None.

## Study approval

This study was reviewed and deemed exempt by the Mayo Clinic IRB, #22-004995.

## Author contributions

Conceptualization: TP, AW, AB. Investigation: KW. Formal analysis: LW, ZL. Writing—original draft: KW. Writing—review and editing: TP, AW, OS, AB. Supervision: TP, AW, AB.

## Patient consent

Informed, written consent was received from all patients for whom photographs are present in the manuscript. All patients involved in the study consented for their medical information and clinical photographs to be published in print and online with the understanding that this information may be publicly available.

## Acknowledgments

The authors extend their gratitude to Victoria Clifton, M.L.I.S. for her assistance with the literature search.
